# The gender gap in obstructive sleep apnea: unmasking the disproportionate costs on women

**DOI:** 10.1093/sleep/zsaf068

**Published:** 2025-04-19

**Authors:** Ahmed S BaHammam

**Affiliations:** Department of Medicine, College of Medicine, University Sleep Disorders Center, King Saud University, Riyadh, Saudi Arabia

Obstructive sleep apnea (OSA) affects approximately 1 billion adults globally, imposing a substantial socioeconomic costs through direct healthcare expenditures and indirect costs [1–3]. Despite growing recognition of the public health significance of OSA, critical knowledge gaps persist regarding gender-specific differences in long-term welfare consequences. While epidemiological studies consistently report higher prevalence in men, emerging evidence suggests women with OSA may experience distinct symptom profiles, diagnostic challenges, and different health trajectories [4].

In their groundbreaking study published in *SLEEP*, Jennum et al. [5] address this knowledge gap through a comprehensive nationwide cohort analysis of 75 024 OSA patients (55 783 men and 19 241 women) compared with controls matched in a 4:1 ratio for age, sex, marital status, and community location. The investigation stands apart through its unprecedented scope, examining both direct health costs and indirect social consequences across an extended observation window that includes up to 15 years before and 11 years after diagnosis, within the 2002–2020 study period. This temporal perspective provides a unique opportunity to understand the evolution of OSA’s impact throughout the course of the disease, including the critical pre-diagnostic period when symptoms may be present but unrecognized.

This editorial contextualizes the findings of Jennum et al. within the broader landscape of OSA research and discusses their far-reaching clinical and policy implications. By highlighting the substantial and disproportionate welfare costs experienced by women with OSA, this study challenges conventional paradigms and underscores the urgent need for gender-sensitive approaches to OSA screening, diagnosis, and management.

Using the Danish National Patient Registry and additional public databases, Jennum and colleagues conducted their analysis on patients aged 20–64 years diagnosed with OSA between 2002 and 2019. Importantly, while patients were identified using the ICD-10 code G47.3 (sleep apnea), >95% specifically had OSA, with diagnoses established through polygraphy or polysomnography according to Danish national standards.

The findings reveal a striking pattern of pre-diagnostic health and social disparities. As early as 15 years before formal diagnosis, individuals later diagnosed with OSA demonstrated significantly higher morbidity across all health domains, greater healthcare utilization, and poorer socioeconomic indicators compared to controls. Following diagnosis, these disparities became more pronounced, with women showing markedly lower employment rates (54.7% vs. 70.4% in controls) compared to men with OSA (74.5% vs. 78.7% in controls), higher rates of disability pension (20.6% vs. 11.1%), and greater case-control net cost differences increasing from €8259 to €13 730 per year for women versus €4217 to €8749 per year for men. Perhaps most compelling was the substantial gender difference observed across all outcomes. Women with OSA experienced significantly higher morbidity, mortality, and social welfare costs than their men counterparts. After adjusting for comorbidities using the Charlson Comorbidity Index, women with OSA showed a 28% higher mortality risk compared to women controls (HR 1.28 [1.20–1.38]), while men with OSA showed only a 3% higher risk that did not reach statistical significance (HR 1.03 [0.99–1.07]). Table 1 summarizes the key findings from Jennum et al., illustrating the profound gender disparities in OSA’s health and socioeconomic costs.

**Table 1.** Gender Disparities in OSA Welfare Consequences: Key Findings From Jennum et al. [5–7].

Parameter	Men with OSA	Women with OSA	Implications
Pre-diagnostic net cost difference (€/year)	4217	8259	Women experience nearly double the economic costs before diagnosis
Post-diagnostic net cost difference (€/year)	8749	13 730	Gender gap persists and widens after diagnosis
Adjusted mortality risk (HR)	1.03 [0.99–1.07]^*^	1.28 [1.20–1.38]	Significant excess mortality in women but not men
Healthcare utilization pattern	Increased	Markedly increased	Women have more healthcare contacts before receiving an OSA diagnosis
Symptom presentation	Classic (snoring, apneas)	Non-specific (fatigue, insomnia)	Different clinical phenotypes require gender-sensitive screening and enhanced clinician awareness
Diagnostic challenges	Less pronounced	More pronounced	Women face greater barriers to accurate diagnosis

^*^Not statistically significant.

The temporal pattern of healthcare utilization is particularly noteworthy, with costs increasing substantially in the years immediately preceding diagnosis—likely reflecting healthcare encounters that eventually led to OSA recognition. This pattern, combined with the extensive pre-diagnostic costs, suggests prolonged periods of undiagnosed disease and missed opportunities for earlier intervention, especially in women who demonstrated consistently higher healthcare utilization yet remained undiagnosed for OSA despite frequent medical contacts.

While Jennum and colleagues present convincing evidence of gender disparities in OSA’s welfare consequences, several methodological considerations warrant discussion. The observed gender differences likely reflect genuine disparities rather than methodological artifacts—supported by consistency across multiple domains and robust statistical adjustments. Nevertheless, registry-based studies have inherent limitations despite high-quality Danish registries [8]. The observational design precludes definitive causal inferences, and several potentially influential confounders remain unmeasured, including body mass index, smoking status, and physical activity levels [9]. The lack of detailed clinical phenotyping represents another significant limitation. Without information on OSA severity parameters, symptom profiles, or treatment adherence, the mechanisms underlying the observed gender differences remain speculative. Recent research suggests that women with OSA may present with distinct clinical phenotypes that could influence both diagnosis and outcomes [6, 9].

While Denmark’s universal healthcare system potentially mitigates financial barriers to diagnosis and treatment, these findings must be interpreted within the context of global healthcare variability. Gender disparities in health outcomes persist across diverse healthcare environments, though their magnitude may vary due to system-specific factors [10]. The gender differences observed likely reflect broader trends seen in other healthcare settings.

The findings provide compelling evidence of substantial OSA underdiagnosis in women and its profound consequences. Despite higher pre-diagnosis healthcare utilization, OSA in women remained unrecognized through numerous medical encounters—a missed opportunity with serious health and economic implications. This pattern aligns with research showing women with OSA face longer diagnostic delays than men [7]. The 28% higher mortality risk in women with OSA compared to women controls underscores the urgency of addressing this diagnostic gap. Gender-specific symptom presentation likely contributes significantly to diagnostic delays. While men typically present with classic symptoms like loud snoring, witnessed apneas, and excessive daytime sleepiness, women more commonly report fatigue, insomnia, morning headaches, and mood disturbances that may be misattributed to other conditions. However, symptomatology alone may not completely explain the diagnostic gap. Other factors, such as socioeconomic factors, healthcare access disparities, and provider bias may also contribute substantially to delayed OSA diagnosis in women [7, 11]. Jennum et al. emphasize that these systemic barriers may further exacerbate gender disparities in OSA recognition. Future research should investigate how these factors collectively impact OSA detection and whether interventions targeting healthcare provider awareness or addressing structural inequities could reduce these disparities [12].

The authors propose several practical approaches to improve OSA identification in women. First, developing screening tools tailored to women’s symptomatology is essential, as current questionnaires were validated predominantly in men. Second, increasing awareness among clinicians, particularly in primary care and gynecology, is crucial. Third, public health campaigns highlighting the diverse symptom profiles of OSA could empower women to recognize potential symptoms and seek appropriate evaluation.

These findings have significant implications for treatment strategies and follow-up care. The higher comorbidity costs and mortality risk in women with OSA suggest they may require more intensive monitoring and comprehensive management approaches. Furthermore, the substantial economic costs—with women’s case–control net cost differences reaching €13 730 per year after diagnosis—highlights the potential economic benefits of earlier intervention [13].

The findings underscore several critical research priorities: (1) prospective studies examining mechanisms underlying gender differences in OSA; (2) development of gender-sensitive screening tools; (3) investigation of gender-specific treatment approaches; (4) economic analyses of early intervention; (5) studies examining OSA’s intersection with hormonal transitions in women [9]; and (6) research into whether “intervention against these inequalities could balance out gender differences” [5]. Implementation of scientific approaches will be crucial to translate these findings into clinical practice.

This landmark nationwide cohort study provides substantial evidence of the disproportionate welfare costs experienced by women with OSA—a finding that demands urgent attention from clinicians, researchers, and policymakers. The striking gender disparities in health outcomes, mortality, and socioeconomic consequences underscore the critical need for increased awareness of the impact of OSA on women. Moving forward, gender-sensitive approaches to screening, diagnosis, and management are essential, including symptom recognition that extends beyond classic presentations, targeted educational initiatives for healthcare providers, and tailored treatment strategies. By addressing these gender disparities through earlier identification and intervention, particularly in women, we could significantly improve health outcomes, reduce healthcare costs, and enhance the quality of life for millions of individuals affected by this highly prevalent but underdiagnosed condition.

## References

[1] Benjafield AV , Ayas NT, Eastwood PR, et al Estimation of the global prevalence and burden of obstructive sleep apnoea: a literature-based analysis. Lancet Respir Med. 2019;7(8):687–698. doi: https://doi.org/10.1016/S2213-2600(19)30198-5 31300334 PMC7007763

[2] Léger D , Stepnowsky C. The economic and societal burden of excessive daytime sleepiness in patients with obstructive sleep apnea. Sleep Med Rev. 2020;51:101275. doi: https://doi.org/10.1016/j.smrv.2020.101275 32169792

[3] Bahammam A , Delaive K, Ronald J, Manfreda J, Roos L, Kryger MH. Health care utilization in males with obstructive sleep apnea syndrome two years after diagnosis and treatment. Sleep. 1999;22(6):740–747. doi: https://doi.org/10.1093/sleep/22.6.740 10505819

[4] Bonsignore MR , Saaresranta T, Riha RL. Sex differences in obstructive sleep apnoea. Eur Respir Rev. 2019;28(154):190030. doi: https://doi.org/10.1183/16000617.0030-2019 31694839 PMC9488655

[5] Jennum P , Ibsen R, Ibsen M, Andersen S, Kjellberg J. Long-term welfare consequences of sleep apnea in 20-64-year-olds - influence of gender: a nationwide cohort study. Sleep. 2025;48(7):1–13. doi:10.1093/sleep/zsaf057 10.1093/sleep/zsaf057 40117453

[6] Romero-Peralta S , García-Rio F, Resano Barrio P, et al Defining the profile of obstructive sleep apnea in women compared to Men. J Womens Health (Larchmt). 2022;31(12):1782–1790. doi: https://doi.org/10.1089/jwh.2021.0659 36166468

[7] Lindberg E , Benediktsdottir B, Franklin KA, et al Women with symptoms of sleep-disordered breathing are less likely to be diagnosed and treated for sleep apnea than men. Sleep Med. 2017;35:17–22. doi: https://doi.org/10.1016/j.sleep.2017.02.032 28619177

[8] Sorensen HT , Sabroe S, Olsen J. A framework for evaluation of secondary data sources for epidemiological research. Int J Epidemiol. 1996;25(2):435–442. doi: https://doi.org/10.1093/ije/25.2.435 9119571

[9] Perger E , Silvestri R, Bonanni E, et al Gender medicine and sleep disorders: from basic science to clinical research. Front Neurol. 2024;15:1392489. doi: https://doi.org/10.3389/fneur.2024.1392489 39050129 PMC11267506

[10] Mauvais-Jarvis F , Bairey Merz N, Barnes PJ, et al Sex and gender: modifiers of health, disease, and medicine. Lancet. 2020;396(10250):565–582. doi: https://doi.org/10.1016/S0140-6736(20)31561-0 32828189 PMC7440877

[11] Lin CM , Davidson TM, Ancoli-Israel S. Gender differences in obstructive sleep apnea and treatment implications. Sleep Med Rev. 2008;12(6):481–496. doi: https://doi.org/10.1016/j.smrv.2007.11.003 18951050 PMC2642982

[12] Moscucci F , Bucciarelli V, Gallina S, et al; Gender Cardiovascular Disease Study Group of the Italian Society of Cardiology (SIC). Obstructive sleep apnea syndrome (OSAS) in women: a forgotten cardiovascular risk factor. Maturitas. 2025;193:108170. doi: https://doi.org/10.1016/j.maturitas.2024.108170 39708590

[13] Streatfeild J , Hillman D, Adams R, Mitchell S, Pezzullo L. Cost-effectiveness of continuous positive airway pressure therapy for obstructive sleep apnea: health care system and societal perspectives. Sleep. 2019;42(12). doi: https://doi.org/10.1093/sleep/zsz181 31403163

